# Multidimensional frailty assessment integrating physical, mental and oral health: a multicentric study

**DOI:** 10.1186/s12903-026-08063-6

**Published:** 2026-03-07

**Authors:** Luciano Maia Alves Ferreira, José Brito, Paulo Alexandre Ferreira Chaló, Carlos Borges Oliveira, Maia e Maia Fischel e Andrade, João Botelho, José João Baltazar Mendes, Evandro Marianeti Fiocco, Simone Cecílio Hallak Regalo

**Affiliations:** 1 Mathias Vitti Electromyography Laboratory, Faculty of Dentistry of Ribeirão Preto, University of São Paulo (FORP/USP), São Paulo, Brazil; 2 Egas Moniz Center for Interdisciplinary Research, Egas Moniz School of Health and Science, Almada, Portugal; 3 Laboratory of Neuroscience, Neuromodulation and Study of Pain (LANNED), Federal University of Alfenas (UNIFAL-MG), Alfenas, Minas Gerais Brazil; 4 Biomechanical and Movement Assessment Laboratory (LABIM), Claretian University Center of Batatais, Batatais, Brazil

**Keywords:** Frailty, Elderly, Oral health, Depression, Bite force

## Abstract

**Aim:**

Frailty is a complex and multidimensional condition commonly observed in older adults and associated with increased risks of falls, hospitalization, disability, and mortality. Traditionally, frailty assessments have focused primarily on physical performance and biomedical parameters. However, emerging evidence highlights the importance of psychological, social, and oral health domains, which are often excluded from standard assessment models. These omissions may limit the accuracy and clinical utility of frailty screening, particularly in diverse aging populations.

**Methods:**

To evaluate the effectiveness of an extended frailty assessment model with up to 10 predictive variables, including Bite force and the Oral Frailty Index (OFI-8/PT), in distinguishing between levels of frailty (non-frail, pre-frail, and frail), and to compare its performance with traditional models.

**Results:**

The sample included 499 community-dwelling older adults (295 from Brazil and 204 from Portugal), aged ≥ 60 years, with at least 20 functional teeth and no cognitive or neurological impairments. Participants underwent functional tests (Timed Up and Go, Sit-to-Stand, handgrip strength), body composition measurements (BMI, body fat percentage, waist-to-hip ratio), psychological assessment (CES-D for depression), physical activity evaluation (IPAQ), Bite force testing (dynamometry), and oral health screening (OFI-8/PT). Discriminant analysis was used to identify which variables best differentiated the frailty groups. Results: In the model with 8 predictors, the main discriminators were the Sit-to-Stand test (STS), handgrip strength, and CES-D depression scores. Discriminant Function 1 (DF1) effectively separated non-frail and frail individuals, while Function 2 (DF2) helped distinguish pre-frail participants. The classification performance of the extended model with 10 variables—adding Bite force and OFI-8/PT—showed no statistically meaningful deterioration, justifying the use of the model with additional predictors of clinical interest. Bite force showed a positive correlation with handgrip strength and a negative correlation with oral frailty scores, indicating its value as an independent functional marker.

**Conclusion:**

Including mental and oral health markers significantly enhances frailty assessment models, supporting a more comprehensive and sensitive approach. The 10-variable model proved effective in identifying distinct frailty profiles, highlighting depression and masticatory function as crucial components. These findings emphasize the need for adaptable, integrative assessment tools in geriatric practice, enabling early, individualized interventions and guiding public health strategies to promote healthy aging across diverse cultural settings.

**Supplementary Information:**

The online version contains supplementary material available at 10.1186/s12903-026-08063-6.

## Introduction

Population aging is a global phenomenon that demands significant transformations in health care systems, social structures, and research priorities. Driven by increased life expectancy and declining fertility rates, this demographic shift has led to a growing proportion of older adults who require innovative strategies to sustain their autonomy, well-being, and quality of life [1]. One of the central challenges in geriatric medicine is frailty, a complex and potentially reversible condition characterized by impaired physiological reserves and high vulnerability to adverse outcomes [2].

Frailty is a multidimensional syndrome encompassing physical, psychological, cognitive, and social components [3], and reflecting cumulative deficits across multiple systems [4]. The widely used frailty phenotype by Fried identifies five indicators: unintentional weight loss, self-reported exhaustion, weakness (grip strength), slowness (gait speed), and low physical activity [5]. While influential, this model does not fully account for psychosocial and oral health, both of which with relevance according to recent discoveries [6].

To address this, the Frailty Index was proposed including accumulation of deficits, including comorbidities, disabilities, and psychosocial risks [7]. Among the less explored domains, oral health is gaining attention. The concept of oral frailty highlights how declines in oral function chewing, speaking, saliva production may signal broader health deterioration [8]. As such, oral frailty was suggested as an early indicator of systemic frailty and malnutrition [9]. Despite its implications, oral health is still often overlooked in frailty screening protocols. Similarly, depression is increasingly recognized as both a contributor to and a consequence of frailty [10]. The bidirectional relationship between physical and psychological frailty suggests that any comprehensive model of frailty must include mental health parameters [6]. Depression scales like the Center for Epidemiologic Studies Depression Scale (CES-D) offer robust tools to capture this dimension.

Technological innovations are expanding the way frailty is measured. The use of biomedical instrumentation, including handgrip dynamometers, surface electromyography (EMG), and bioimpedance analyzers, allows clinicians to collect objective and quantifiable data. These tools provide precise data that improve clinical decision making and individualized interventions [9], using bite force as a new functional marker. The bite force is also consensually associated with general muscle strength and fragility phenotype, regardless of age and BMI [10]. This study aimed to analyze the effectiveness of an expanded frailty assessment model, composed of traditional physical markers, mental health parameters (depression), and the incremental contribution of oral health indicators (including bite strength and oral frailty), in identifying and distinguishing between different stages of frailty in community-dwelling older adults in Brazil and Portugal. Specifically, it sought to compare the discriminatory power of this new multidimensional model with the traditional model, based exclusively on physical indicators, to determine which one classifies frailty stages more effectively.

## Methods

The present cross-sectional study is reported following the ‘Strengthening the Reporting of Observational studies in Epidemiology’ (STROBE) checklist [11]. The present study was carried out following the Declaration of Helsinki, updated in 2024, approved by the Egas Moniz Ethical Committee (research number PT-348/23) and by Research Ethics Committee of the Faculty of Dentistry of the University of São Paulo (nº 6.787.851, CAAE: 79222024.8.0000.5419, on April 25, 2024).

### Design and setting

Participants were recruited from two community-based health programs from two different countries: the “*Sempre a Mexer*” program in Sesimbra (southern area of the Lisbon Metropolitan Area, Portugal) and CASI Project (*Centro de Apoio e Saúde do Idoso*) in Batatais, Brazil).

In this study, subjects were recruited following a consecutive convenient sampling between April and June 2024 for the Portuguese cohort, and between July and September 2024 and signed an informed consent.

### Eligibility criteria and participants

Patients were included if: aged 60 or older; being actively engaged in at least 150 min of physical activity per week; with at least 20 functional teeth to allow accurate Bite force readings [12]; and capable of independent living with appropriate cognitive and oral function. Patients were excluded if presented clinical diagnosis of dementia-related neurological conditions.

### Assessment tools

Both quantitative and qualitative assessment tools were used to comprehensively evaluate the participants’ physical, functional, and oral health characteristics. Quantitative: Body Composition measured with Bioimpedance (Accuniq BC300), EMG System’s bite dynamometer, and KINVENT grip and forceps dynamometers. Qualitative: Timed Up and Go Test (TUGT): Measures mobility and dependence on daily activities [13, 14], Sit-to-Stand Test (STS): Assesses lower limb function, balance, and mobility [15], Handgrip Strength: Maximum grip force maintained for 6 s [16], Bite Force: Maximum force applied during a 6-second bite, recorded using EMG and dynamometry [17], Oral Frailty Index (OFI): Assesses frailty related to oral health [18, 19], Center for Epidemiologic Studies Depression Scale (CES-D): Screens for depression symptoms [20] and International Physical Activity Questionnaire (IPAQ): Evaluates physical activity levels [21].

### Protocol

The protocol established a rigorous calibration and team training process, requiring all assessors to undergo 4 h of instruction and achieve a minimum Intraclass Correlation Coefficient (ICC) of 0.85 in pilot tests to ensure measurement uniformity. Calibration of the EMG System (bite) and KINVENT (grip) dynamometers was performed weekly, and calibration of the Accuniq BC300 bioimpedance equipment was performed daily (zero point verification). Assessments of participants, primarily elderly individuals, were scheduled for the morning (9:00–12:00) and conducted in a quiet environment, with crucial instructions regarding fasting and abstinence from physical exercise for 4 h prior to the test, essential for bioimpedance analysis. The fixed order of tests was maintained (OFI-8/PT, CES-D, IPAQ questionnaires; bioimpedance; strength; performance). Although the strength assessments involved multiple attempts, only one final value was recorded for analysis: for Hand Grip Strength, the maximum value for each hand was recorded in three attempts; for the bite strength test, the maximum (peak) value was recorded in three attempts; and for the Sit-to-Stand (STS) and Timed Stand and Walk (TUGT) performance tests, the repetition count or execution time was recorded as the final result of the functional assessment.

### Frailty index

The Frailty Index classification consists of three groups: G0 (Non-frail), with no positive criteria; G1 (Pre-frail), with one positive criterion; and G2 (Frail), with two or more positive criteria. The first model (“*Eight-predictor model”)* includes the following variables: Grip Strength (Right and Left), Body Mass Index (BMI), Body Fat Percentage (FAT%), Waist-to-Hip Ratio (WHR), Timed Up and Go Test (TUGT), Sit-to-Stand (STS), and the Center for Epidemiologic Studies Depression Scale (CES-D). Following initial analysis, Bite force and the Oral Frailty Index (OFI-8) were added to the model to further refine the assessment *(Ten-predictor model”).*

### Statistical analysis

Missing data were minimal across variables. Overall, *n* = 54 participants (9.8%) were excluded due to incomplete data ([Brazil: *n* = 1, 0.3%]; [Portugal: *n* = 54, 20.6%]). Because the proportion of missingness was low and consistent with a Missing Completely at Random (MCAR) pattern, analyses were conducted using a complete-case approach, which was considered unlikely to meaningfully bias estimates or reduce statistical power.

Discriminant Analysis (DA) was used to identify which physical, psychological, and oral health variables are the most relevant predictors for discriminating between frailty status groups. Analyses were conducted separately for Brazilian and Portuguese cohorts. Discriminant Analysis was performed after checking the model’s assumptions, namely normal distribution of the predictor variables and homogeneity of the variance-covariance matrix. The univariate normal distribution of each variable was tested using with the Shapiro-Wilk test, which excluded multivariate normality for most predictor variables. The M-Box test for homogeneity of variance-covariance matrices has also revealed a violation of such assumption. However, DA is relatively robust to violations of multivariate normality and the homogeneity of variance-covariance matrices when the sample sizes are reasonably large, particularly for the smallest group and the number of cases in the smallest group exceeds the number of independent variables, as is the case in this study. In addition, the lack of proportionality between means and variances observed across groups in this study make DA less sensitive to violations of homogeneity of variance-covariance matrices. In sum, DA can still provide meaningful results when assumptions are violated, since group sizes are sufficient, and means and variances are not proportional. All statistical procedures were implemented using IBM SPSS Statistics for Windows, Version 29.0.2.0 Armonk, NY: IBM Corp.

We have used MANOVA as a surrogate framework for sample size estimation for Discriminant Analysis (DA) within GPower. By modelling 10 dependent variables in the MANOVA, the multidimensional structure expected in the DA is approximated, using GPower’s MANOVA: Global effects procedure to determine the necessary sample size. The anticipated multivariate effects are low, with Pillai’s trace values corresponding roughly to 0.11 for small to moderate effects (effect size = f^2^(V) = 0.058). With an 80% target power and a 5% significance level, G*Power computed a total sample size of 189 to be distributed across the three groups.

In the event of cases having missing data strategy, the decision was to remove such cases from the final analysis.

## Results

### Participants characteristics

From an initial sample of 553 participants (296 from Brazil and 257 from Portugal), a total of 499 older adults were included in the analysis (295 participants from Brazil and 204 from Portugal). The excluded participants were due to lack of the minimum number of teeth present. Sex distributions were similar in both cohorts: in the Brazil group, there were 234 (79.3%) female and 61 (20.7%) participants, whereas the Portugal group included 167 (81.9%) female and 37 (18.1%) male participants. Sex distribution per region and frailty group is detailed in Supplementary Tables S1 and S2.

Both cohorts had comparable mean ages of 72.6 and 73.7 years, respectively (Table 1). Both groups exhibited similar patterns across physical and psychosocial variables. Mean grip strength was slightly higher in Brazil for both right (17.1 kgf) and left (16.7 kgf) hands, compared to Portugal (16.6 kgf and 16.2 kgf, respectively). Depressive symptoms, assessed using the CES-D scale, averaged 12.9 in Brazil and 13.4 in Portugal. Functional mobility, measured by the Timed Up and Go (TUG) test, was slower in Brazil (10.3 s) than in Portugal (7.6 s), suggesting better mobility among Portuguese participants. Oral frailty scores (OFI-8/PT) were similar across both groups (mean = 4.5). Portuguese participants had a slightly lower mean body fat percentage (34.5%) than Brazilians (36.7%), while BMI values were only reported for the Brazilian cohort (mean = 28.4 kg/m²). Bite force, a novel marker of physical and oral function, was marginally higher in Brazil (18.0 kgf) compared to Portugal (data not available).

**Table 1:** Descriptive statistics

Region		*N*	Min	Max	Mean	SD
Brazil	Age	295	55.0	92.0	72.6	6.5
	Rigth Grip Strength (Kgf)	295	4.5	43.9	17.1	6.0
	CES-D	295	0.0	42.0	12.9	9.1
	TUG (seg)	295	5.0	27.4	10.3	2.9
	OFI-8/PT	295	1.0	11.0	4.5	2.2
	FAT (%)	295	3.0	60.0	36.7	11.0
	STS (rep)	295	4.0	24.0	11.1	3.3
	Left Grip Strength (Kgf)	295	5.8	39.7	16.7	6.0
	Bite force (Kgf)	295	2.0	61.2	18.0	7.7
	BMI (kg/m2)	295	14.6	44.0	28.4	5.3
	WHR (w/r)	295	0.4	1.1	0.9	0.1
Portugal	Age	204	56.0	92.0	73.7	6.6
	Rigth Grip Strength (Kgf)	204	6.8	40.2	16.6	5.0
	CES-D	204	0.0	42.0	13.4	9.1
	TUG (seg)	204	4.4	17.8	7.6	1.9
	OFI-8/PT	204	1.0	10.0	4.5	2.0
	FAT (%)	204	7.0	52.1	34.5	6.4
	STS (rep)	204	6.0	30.0	16.3	3.8
	Left Grip Strength (Kgf)	204	5.1	33.8	16.2	4.8
	Bite force (Kgf)	204	2.9	35.4	20.1	7.9
	BMI (kg/m2)	204	18.2	45.6	27.4	4.4
	WHR (w/r)	204	0.7	1.2	0.9	0.1

Participants were categorized into three frailty groups based on adapted Fried criteria: non-frail, pre-frail, and frail. Discriminate analysis was used to evaluate the ability of physical, psychological, and oral health variables to predict frailty status.

Table 1 presents descriptive statistics of the study variables and includes demographic data, physical health indicators and frailty-related measures, mean, standard deviation (SD), minimum (Min) and maximum (Max) values ​​for each variable in the Brazilian (295 individuals) and Portuguese (204 individuals) samples.

### Discriminant analysis

Supplementary Tables S1 and S2 provide detailed descriptive statistics data used in the diagnostics of assumptions for Discriminant Analysis using the “Eight-predictor model”. Based on the combined evidence from the univariate and multivariate tests, the assumption of multivariate normality is clearly violated. Several variables fail the Shapiro–Wilk test (Supplementary Table S3), which alone excludes the possibility of a joint multivariate normal distribution. This conclusion is reinforced by the multivariate diagnostics: Mardia’s skewness and kurtosis statistics are significant in most fragility groups in both cohorts, and the Henze–Zirkler test likewise rejects multivariate normality (Table 2).

**Table 2:** Multivariate normality tests *(“Eight-predictor model”)*

Multivariate Normality Tests					
		Brazil		Portugal	
Fragility Group	Test	Statistic	sig.	Statistic	sig.
0	Henze-Zirkler	0.970	0.085	1.248	< 0.001
	Mardia Skewness	161.279	0.007	210.904	< 0.001
	Mardia Kurtosis	0.296	0.767	3.083	0.002
1	Henze-Zirkler	1.577	< 0.001	1.405	< 0.001
	Mardia Skewness	478.625	< 0.001	314.141	< 0.001
	Mardia Kurtosis	7.866	< 0.001	5.331	< 0.001
2	Henze-Zirkler	1.166	< 0.001	1.191	< 0.001
	Mardia Skewness	307.049	< 0.001	497.044	< 0.001
	Mardia Kurtosis	5.285	< 0.001	11.327	< 0.001

Multivariate outliers were identified using adjusted Mahalanobis distance and tested against group-specific chi-square thresholds (df = 8). Most flagged cases per group in each cohort were statistically significant. Taken together, these results demonstrate that the data deviate substantially from the assumptions required for multivariate normality. Moreover, Box’s M test indicated significant results in both cohorts, shown in Table 3 confirming that the assumption of homogeneity of covariance matrices was violated.

**Table 3:** Box’s test of covariance matrices homogeneity *(“Eight-predictor model”)*

	Covariance Matrices Homogeneity Tests Results		
	Box’s M	Brazil	Portugal
		220.6	226.6
F	Approx.	2.9	3.0
	df1	72.0	72.0
	df2	31988.2	100091.0
	Sig.	< 0.001	< 0.001

Multicollinearity was formally tested using variance inflation factors (VIF) in Multiple Regression Models where each of the independent variables in the Discriminant Analysis was regressed against the remaining 7 variables as predictors. All VIF values among these predictors were below 5 (Supplementary Tables S4 and S5), confirming that collinearity among the eight independent variables in the Discriminant Analysis was not problematic.

**Fig. 1:**
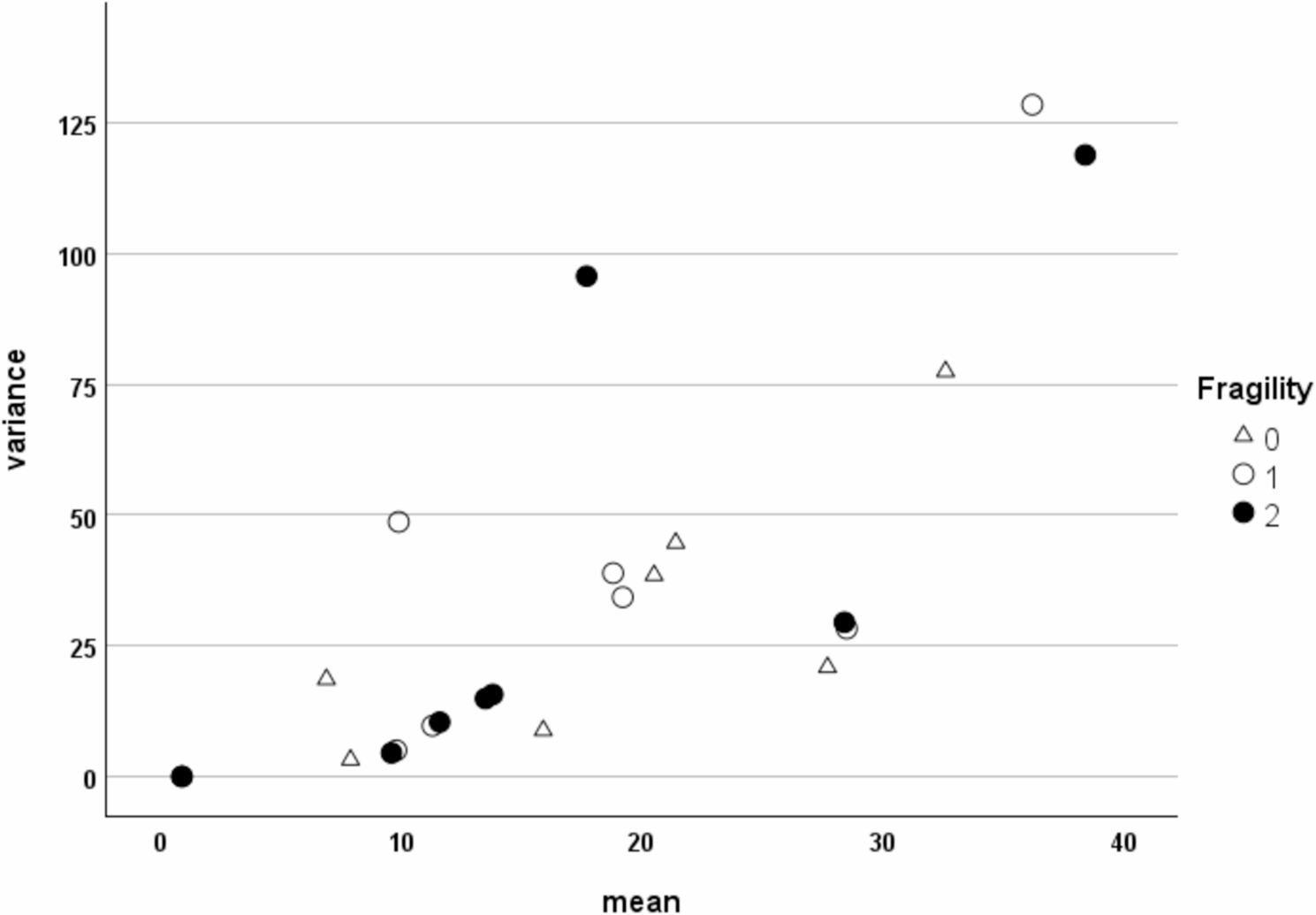
Scatter plot of variances versus means of the independent variables, cohort Brazil (*“Eight-predictor model”*)

Despite violations of multivariate normality and homogeneity of covariance matrices, discriminant analysis remains viable in this study. Two key conditions are met following Pituch and Stevens (2015) [22]: first, the number of variables (eight) is smaller than the size of the smallest group in each cohort (*N* = 35, in Brazil; *N* = 57, in Portugal), ensuring sufficient dimensional support for the model; second, the proportionality between group means and variances is explicitly violated, as demonstrated by the scatter plots in Fig. 1 and Supplementary Fig. 1. In both cohorts, the relationship between mean and variance does not follow a consistent scaling pattern across groups. The dispersion of data points by fragility level shows that variance does not increase uniformly with the mean. This lack of proportionality reinforces the justification for applying discriminant analysis under relaxed assumptions, as supported by the literature. The method is therefore retained, with results interpreted cautiously and transparently.

For the expanded “Ten-predictor model”, all discriminant analysis assumptions were checked using the same diagnostic procedures applied to the “Eight-predictor model”). The violations of multivariate normality and homogeneity of covariance matrices were also observed with the expanded model, but their magnitude do not undermine model application as discussed above for the “Eight-predictor model”, and based on the results shown in Supplementary Tables S7 – S10, and Supplementary Figs. 2 and 3. Therefore only the key performance indicators (canonical correlations and classification metrics) are reported here.

### Cohort from Brazil

The adjusted discriminant model resulted in eight strongly correlated predictors to differentiate the frailty status. Discriminant Function (DF) 1 accounted for most of the discriminatory power (Wilk’s Lambda = 0.395, chi-square 267.767, GL (DF) of 16, and *p* < 0.001), with a canonical correlation of 0.751. As for DF2, it is statistically significant but contributed less to overall discrimination (Wilk’s Lambda = 0.905, chi-square 28.744, GL (DF) of 7, and *p* < 0.001), with a canonical correlation of 0.308.

In the structure matrix analysis (Table 2) for predictor relevance, key predictors in DF1 were STS (λ = 0.607), Right Grip Strength (λ = 0.481), Left Grip Strength (λ = 0.449), and CES-D (λ = -0.447). Key predictors in DF2 were STS (λ = 0.698), Left Grip Strength (λ = -0.489), and CES-D (λ = 0.460). WHR and BMI were less relevant predictors, having consistently low correlations with both functions and indicating minimal contribution to group differentiation.

Regarding frailty group differentiation (Table 3), DF1 mainly separates Group 0 (Controls) from Group 2 (Frail). Group 1 (Pre-frail) is intermediate and less distinct along this function. In addition, DF2 helps differentiate Group 1 (Prefrail) from Groups 0 (Controls) and 2 (Frail), although the separation is less pronounced compared to DF1. In short, DF1 is the dominant function, explaining most of the variance and providing strong discrimination between Groups 0 and 2. DF2 adds nuance by differentiating Group 1 from the other two groups (Table 4).

**Table 4:** Structure matrix *(“Eight-predictor model”)*

Variable	Function 1	Function 2
Right Grip Strength (Kgf)	0.481*	-0.466
TUG (seg)	-0.402*	-0.009
FAT (%)	-0.148*	-0.069
STS (rep)	0.607	0.698*
Left Grip Strength (Kgf)	0.449	-0.489*
CES-D	-0.447	0.460*
BMI (kg/m²)	-0.019	-0.131*
WHR (w/y)	-0.005	-0.088*

Brazil

Within-group correlations between discriminatory variables and standardized canonical discriminant functions

^a^Variables ordered by the absolute size of the correlation function with the function

^*^Highest absolute correlation of each variable with any discriminant function

Classification performance for this cohort was satisfactory, based on the analysis of the confusion matrix (Table 5). The apparent accuracy of the discriminant analysis was 73.6%. while the cross-validated accuracy was 71.5%. indicating only a modest reduction under validation. Per-group sensitivities were 74.3% for Group 0. 66.9% for Group (1) and 75.6% for Group (2) Corresponding specificities were 91.2%. 75.9%. and 87.0%. respectively. Confidence intervals around these estimates confirmed their stability: for example. Group 1 sensitivity was 66.9% (95% CI: 58.5–74.3%). and Group 1 specificity was 75.9% (95% CI: 68.8–81.9%). Overall agreement between predicted and actual classifications was moderate. with a Cohen’s kappa of 0.54 (95% CI: 0.45–0.62). These results demonstrate consistent classification performance across groups. with uncertainty estimates reported for the sake of transparency.

**Table 5:** Confusion matrix for cohort Brazil *(“Eight-predictor model”)*. Cross-validation using the leave-one-out method (“U-method”)

Classification Results						
Fragilidade			Predicted Group Membership			Total
			0	1	2	
Original	Count	0	26	9	0	35
		1	21	93	19	133
		2	0	29	98	127
	%	0	74.3	25.7	0.0	100.0
		1	15.8	69.9	14.3	100.0
		2	0.0	22.8	77.2	100.0
Cross-validated	Count	0	26	9	0	35
		1	22	89	22	133
		2	1	30	96	127
	%	0	74.3	25.7	0.0	100.0
		1	16.5	66.9	16.5	100.0
		2	0.8	23.6	75.6	100.0

### Cohort from Portugal

The adjusted discriminant model resulted in eight strongly correlated predictors to differentiate the frailty status. Discriminant Function (DF) 1 accounted for most of the discriminatory power (Wilk’s Lambda = 0.300, chi-square 238.092, GL (DF) of 16, and *p* < 0.001), with a canonical correlation of 0.823. As for DF2, it was statistically significant but contributed less to overall discrimination (Wilk’s Lambda = 0.927, chi-square 15.019, GL (DF) of 7, and *p* = 0.036), with a canonical correlation of 0.271.

In the structure matrix analysis (Table 4) for predictor relevance, key predictors in DF1 were CES-D (λ = 0.690), Right Grip Strength (λ = -0.430); Left grip strength (λ = -0.415); STS (λ = -0.315), and TUG (λ = 0.254). Key predictors in DF2 were Right Grip Strength (λ = 0.796); Left grip strength (λ = 0.721); FAT (λ = -0.429), and CES-D to a lesser extent (λ = 0.428). WHR and BMI were less relevant predictors, having consistently low correlations with both functions and indicating minimal contribution to group differentiation.

Regarding frailty group differentiation (Table 3), DF1 mainly separates Group 0 (Controls) from Group 2 (Frail). Group 1 (Pre-frail) is intermediate and less distinct along this function. In addition, DF2 helps differentiate Group 1 (Prefrail) from Groups 0 (Controls) and 2 (Frail), although the separation is less pronounced compared to DF1. In short, DF1 is the dominant function, explaining most of the variance and providing strong discrimination between Groups 0 and 2. DF2 adds nuance by differentiating Group 1 from the other two groups (Table 6).

**Table 6:** Structure matrix (“*Eight-predictor model*”)

Variable	Function 1	Function 2
CES-D	0.690*	0.428
STS (rep)	-0.315*	-0.113
TUG (seg)	0.254*	-0.094
BMI (kg/m²)	0.113*	0.047
WHR (w/y)	-0.066*	0
Right Grip Strength (Kgf)	-0.430	0.796*
Left Grip Strength (Kgf)	-0.415	0.721*
FAT (%)	0.192	-0.429*

Portugal

^a^Within-group correlations between discriminatory variables and standardized canonical discriminant functions. Variables ordered by the absolute size of the correlation function with the function

^*^Highest absolute correlation of each variable with any discriminant function

Regarding frailty group differentiation (Table 7), Function 1 (DF1) primarily separates Group 0 (Controls) from Group 2 (Frail), with mean values of -1.479 and 2.102, respectively. Group 1 (Pre-frail), with a mean of -0.084 on DF1, lies in between and is less distinct along this function. Additionally, Function 2 (DF2) contributes modestly to the separation, particularly between Group 1 (Pre-frail), which scores − 0.386, and the other two groups, which are closer together (0.214 for Controls and 0.184 for Frail) (Table 8). In summary, DF1 is the dominant discriminant function, offering strong differentiation between Controls and Frail individuals, while DF2 provides secondary discrimination by distinguishing the Pre-frail group from the others.

**Table 7:** Functions at groupcentroids. *(“Eight-predictor model”)*

Fragility	Function 1	Function 2
0	2.254	0.598
1	0.498	-0.326
2	-1.143	0.176

Non-standard canonical discriminant functions evaluated in group settings

**Table 8:** Functions at group centroids ͣ (“*Eight-predictor model*”)

Fragility	Function 1	Function 2
0	-1.479	0.214
1	-0.084	-0.386
2	2.102	0.184

Non-standard canonical discriminant functions evaluated in group means

For cohort Portugal. classification performance was again satisfactory, based on the confusion matrix (Table 9). The apparent accuracy of the discriminant analysis was 75.5%. while the cross-validated accuracy was 72.1%. showing only a modest reduction under validation. Per-group sensitivities were 68.8% for Group 0. 67.1% for Group (1) and 82.5% for Group (2) Corresponding specificities were 85.0%. 75.4%. and 96.6%. respectively. Confidence intervals confirmed the robustness of these estimates: for example. Group 0 sensitivity was 68.8% (95% CI: 57.8–78.1%). and Group 2 specificity was 96.6% (95% CI: 92.2–98.6%). Overall agreement between predicted and actual classifications was moderate. with a Cohen’s kappa of 0.58 (95% CI: 0.49–0.66). Again, these results demonstrate consistent classification performance across groups in this cohort.

**Table 9:** Confusion matrix for cohort Portugal (“Eight-predictor model”). Cross-validation using the method leave-one-out (“U-method”)

Classification Results						
*Frailty*			*Predicted Group Membership*			*Total*
			*0*	*1*	*2*	
Original	Count	0	56	21	0	77
		1	15	50	5	70
		2	0	9	48	57
	%	0	72.7	27.3	0.0	100.0
		1	21.4	71.4	7.1	100.0
		2	0.0	15.8	84.2	100.0
Cross-validated	Count	0	53	24	0	77
		1	18	47	5	70
		2	1	9	47	57
	%	0	68.8	31.2	0.0	100.0
		1	25.7	67.1	7.1	100.0
		2	1.8	15.8	82.5	100.0

### Assessment of sex and age as potential confounders

To evaluate the potential influence of sex and age on the discriminant analyses, we first examined whether these variables differed across frailty groups in each cohort. ANOVA tests indicated no significant differences in mean age between frailty groups in the Brazil (F = 0.908; *p* = 0.406) and Portugal (F = 0.982; *p* = 0.376) group, suggesting that age does not act as a confounding factor. In contrast, chi-square tests revealed that sex was associated with frailty group membership in both cohorts (*p* < 0.001 in both cases), and subsequent analyses therefore focused on sex as the variable of concern.

Sex (dummy coded) was then included as a predictor in the discriminant analyses. In the Brazil cohort, the addition of sex did not alter the magnitude or significance of the canonical correlations, the statistical significance of the discriminant functions, the set of relevant predictors, or the classification accuracy (sensitivity and specificity unchanged). In the Portugal cohort, results were identical except that sex loaded on discriminant function 2, which accounted for only ~ 5% of discriminant power. Overall, these findings indicate that sex does not materially influence the discriminant functions, and the models remain robust to potential sex imbalance.

### Inclusion of ‘Bite force’ and OFI8-PT as predictors

The inclusion of Bite force and OFI-8/PT in the discriminant analysis, now a 10-predictor model, was justified by bivariate correlations with discriminant functions. Discriminant function analysis revealed statistically significant results in both regions. In the Brazilian cohort, the combined model (Functions 1 and 2) demonstrated strong discriminatory power, with a Wilks’ Lambda of 0.386 and a highly significant Chi-square value (χ² = 274.018, df = 20, *p* < 0.001), with a canonical correlation of 0.752. Function 2 alone also showed a significant contribution (*p* < 0.001), albeit with a higher Wilks’ Lambda (0.888), with a canonical correlation of 0.335, indicating a more modest effect. In the Portuguese cohort, the overall model (Functions 1 and 2) also yielded significant discrimination (Wilks’ Lambda = 0.297, χ² = 238.321, df = 20, *p* < 0.001), with a canonical correlation of 0.823. However, Function 2 by itself did not reach statistical significance (*p* = 0.072), with a Wilks’ Lambda of 0.923, a canonical correlation of 0.278, suggesting that the discriminatory power in Portugal was driven primarily by Function 1.

The structure matrix for both cohorts (Table 10) revealed the variables most strongly associated with each discriminant function for both cohorts. In the Brazilian cohort, Function 1 (DF1) was primarily characterized by positive loadings of STS repetitions (0.606), right grip strength (0.477), and left grip strength (0.445), indicating that muscular strength and physical performance were key discriminators. CES-D and TUG scores showed moderate negative loadings on DF1, reflecting an inverse relationship between depressive symptoms, mobility limitations, and physical robustness. Function 2 (DF2) was also strongly influenced by STS repetitions (0.610), followed by negative associations with left grip strength (-0.464) and right grip strength (-0.445), suggesting a distinct dimension of physical frailty. In contrast, the Portuguese cohort showed a slightly different pattern. DF1 was dominated by negative correlations with CES-D (-0.689), left grip strength (-0.707), and right grip strength (-0.428), suggesting that emotional and physical components aligned in defining the primary discriminant function. On DF2, the strongest loading was observed for OFI-8/PT (0.241) and FAT% (0.420), indicating that oral frailty and body composition contributed more to the second discriminant dimension in this population. Notably, STS repetitions contributed moderately to DF1 (0.314) in Portugal, reinforcing its relevance across contexts. Overall, muscular strength and emotional well-being were the most discriminative features in both cohorts, though the weight and direction of their contributions varied between countries.

**Table 10:** Structure matrix of the Brazilian (*n* = 295) and the Portuguese (*n* = 204) cohorts (“Ten-predictor model”)

Variable	Brazilian cohort (*n* = 295)		Portuguese cohort (*n* = 204)	
	DF1	DF2	DF1	DF2
Right Grip Strength (Kgf)	0.477	-0.445	0.428	-0.780*
CES-D	-0.443	0.437	-0.689*	-0.405
TUG (seg)	-0.400	0.009	-0.254*	0.095
OFI-8/EN	-0.219	-0.095	-0.214	0.241*
FAT (%)	-0.148	-0.056	-0.191	0.420*
STS (rep)	0.606	0.610	0.314*	0.105
Left Grip Strength (Kgf)	0.445	-0.464	0.414	-0.707*
Bite force (Kgf)	0.137	-0.369	0.155*	-0.139
BMI (kg/m²)	-0.02	-0.119*	-0.112*	-0.044
WHR (w/y)	-0.005	-0.08*	0.066*	-0.001

The variable with the highest absolute value of correlation in each function is marked with an asterisk (*). The matrix represents the within-group correlations between discriminant variables and standardized discriminant functions. Variables are ordered by the absolute size of the correlation with function 1

We have conducted a performance comparison between the two models in each cohort, based on the confusion matrices. Supplementary Table S6 gathers the main results of that comparison. Across both cohorts, the expanded ten-predictor models did not yield substantive gains in classification performance relative to the eight-predictor models. In Brazil, sensitivities were modestly lower across all groups (by 2–3% points), while Cohen’s κ remained unchanged. In Portugal, sensitivities for Groups 0 and 2 were identical, with Group 1 showing only a marginal reduction, and κ again essentially stable. Importantly, all differences fell within overlapping confidence intervals, indicating no statistically meaningful deterioration. Thus, while parsimony favors the eight-predictor models, the expanded ten-predictor models remain clinically defensible: they maintain stable accuracy and agreement, and their use is justified since there is clinical interest in incorporating the additional predictors.

## Discussion

Early identification of frailty in older adults is essential to prevent complications and guide appropriate interventions. The model proposed by Fried et al. remains one of the most widely used references, based on five physical criteria: involuntary weight loss, fatigue, decreased handgrip strength, slow walking speed, and low physical activity. However, this approach, which focuses primarily on physical indicators, may not fully capture the multidimensional nature of frailty. Importantly, even among active individuals without significant impairments, the results indicate that frailty risk is present, underscoring the need for monitoring and early detection even in seemingly healthy populations. In this study, models with 8 and 10 predictor variables were compared to Fried’s traditional model, through discriminant analyses applied to two different samples, from Brazil and Portugal. The results indicated that the first discriminant function (DF1) accounted for most of the discriminatory variance, with a significant reduction in Wilks’ Lambda and acceptable separation between individuals classified as Controls and Frail. The second discriminant function (DF2), although with less explanatory weight, was also statistically significant, being especially relevant to differentiate the Pre-fragile group, an intermediate and potentially reversible stage of the syndrome.

By integrating oral health and mental well-being into frailty assessments, we aim to promote a more holistic understanding of aging. As stated by Dibello et al. [23], multidimensional tools are essential to detect early signs of frailty and guide personalized interventions. Our findings can contribute to refining diagnostic protocols and informing public health strategies for aging populations.

The discriminant analysis using the model with eight predictors revealed a comprehensive understanding of the factors associated with frailty in the elderly, considering the interaction between physical, mental and body composition aspects.

Although the literature already recognizes frailty as a multifactorial geriatric condition characterized by vulnerability to stressors and higher risk of morbidity, disability, and mortality, there is still a lack of instruments that integrate anthropometric measures and other relevant dimensions, such as oral health and psychosocial factors [24, 25].

In the Brazilian cohort, the main predictors were the sit-and-stand test (STS), handgrip strength (Grip Strength), and the CES-D score, reflecting the relationship between physical performance, mental health, and frailty, corroborating the literature that associates physical performance and mental health with frailty [26, 27]. In the Portuguese sample, the TUG test and body fat percentage (FAT) were also highlighted, reinforcing a broader approach, in line with the multidimensional perspective defended by Rockwood et al., which incorporated physical and psychological deficits in the concept of frailty. The inclusion of variables such as Bite force and the OFI-8/PT index in the model with 10 predictors sought more specific dimensions of frailty, such as oral function and general health, in line with recent studies on the subject [27, 28].

It is important to highlight that the use of the CES-D scale allowed the identification of depressive symptoms as influences of the condition of frailty, in addition to its original use in the assessment of exhaustion. The data revealed an association between depressive signs and factors such as loss of muscle strength, worse functional performance, and negative perception of oral health. These results agree with the literature that points to depression as an aggravating factor of physical vulnerability, in a feedback process: physical limitations intensify social isolation, while apathy resulting from depression compromises the adoption of healthy habits [29].

In addition, studies have shown that instruments such as the Edmonton Frail Scale are effective in assessing frailty more broadly, including aspects such as cognition, mood, and social support [30].

In both cohorts analyzed, DF1 provided the strongest discrimination between the Control and Frail groups, while DF2, despite a lower statistical contribution, helped differentiate nuances within the Pre-frail group. These findings support the potential value of na integrated assessment of frailty that considers physical, psychological and social dimensions, which may help inform future preventive and supportive strategies [31].

Expanding the model to include 10 predictors specifically, the OFI-8/PT index and Bite force resulted in a slight decrease in classification performance which did not reach statistically meaningful deterioration but added practical value to its use with predictors of clinical interest. These added predictors were associated with the discriminant functions, underscoring the important role of oral function in assessing frailty.

The oral frailty index (OFI-8) suggests sensitivity in identifying nutritional risks, since higher scores were associated with higher consumption of pasty foods. These results reinforce international guidelines that advocate the incorporation of oral health in geriatric evaluation, considering that masticatory changes can precede and accelerate functional decline [19, 25].

The study by Dibello et al. enriches the discussion by highlighting the connection between frailty and oral health. Research points out that oral weakness is common in the elderly and is associated with deterioration of oral health and reduced oral motor skills, negatively impacting functionality and increasing the risk of physical and nutritional frailty [32].

The average bite force observed was lower than that of young adults among elderly individuals of both sexes [18]. Assessments performed with a dynamometer showed that bite force correlates positively with the presence of functional teeth and negatively with indicators of oral fragility, standing out as a promising functional marker for assessing fragility, as evidenced in the study by Ferreira et al., which identified a positive correlation between bite force and handgrip strength, even after controlling for age and BMI, suggesting that it is an independent functional indicator [10]. The integration of the instruments used allowed the identification of three distinct profiles of frailty among the elderly: one with a predominance of physical-metabolic characteristics, another focused on psycho-functional aspects, and a third with emphasis on oral health and nutritional status. This typification points to the importance of individualized strategies, such as strength exercises for the physical-metabolic profile, protein supplementation for oro-nutritional cases, and psychological support for individuals with psycho-functional frailty.

### Principal results

Thus, the model with 10 predictors provides a broader understanding of frailty by incorporating variables from different domains. Discriminant analysis showed acceptable discrimination between groups and helped identify relevant predictors within this selected sample. The recurrence of CES-D as a relevant predictor in both samples reinforces the strong link between mental health and physical frailty. The results reinforce that oral health should be an integral part of frailty prevention strategies, within a truly multidimensional approach to geriatric care.

### Limitations

The study has limitations, such as the sample being restricted to older adults with at least 20 functional teeth, excluding those with significant tooth loss or edentulous individuals, which may reduce the generalizability of the results. Additionally, the exclusion of individuals with dementia or neurological pathologies and the lack of control over variables such as comorbidities and medications could impact the assessments. Cross-sectional design also prevents causal conclusions, and the use of subjective measures, such as self-reported questionnaires, may introduce bias. The specialized technology used limits the study’s replicability, and cultural differences between Brazil and Portugal may affect comparisons with other contexts.

Moreover, although procedural aspects of bite-force assessment (calibration, assessor training, test order, and three repeated attempts with peak-value recording) were standardized and described in the Methods, some relevant clinical details regarding dentition were not collected. Specifically, data on the exact number and distribution of teeth present, as well as the presence or type of dental prostheses, were not recorded, which may influence masticatory performance and bite-force values. In addition, jaw-position standardization was based on functional instructions rather than instrumented or occlusal-registration methods, which may limit measurement precision. These factors may affect the interpretation and generalizability of oral-function findings. Future studies should incorporate more detailed dental characterization and standardized biomechanical positioning protocols to enhance reproducibility and external validity.

### Comparison with previous work

The current study builds on established frailty models but adopts a more comprehensive and integrative approach, combining measures of physical, psychological and oral health. The use of two cohorts also demonstrates the caution that should be taken in interpreting frailty based on general phenotypes. The inclusion of bite force, oral frailty and emotional aspects differentiates this study from previous research, making it a valuable contribution to the understanding of frailty in older adults.

## Conclusions

This study suggests that a multidimensional model integrating physical, emotional, and oral health variables may improve the classification of frailty stages among older adults in Brazil and Portugal within this sample. The first discriminant function distinguished Controls from Frail individuals, while the second aided identification of Pre-frail cases. Bite force and oral frailty provided additional discriminatory information. The 10-variable model showed acceptable classification performance, and cross-country differences highlighted the importance of context-sensitive assessment. Overall, these findings indicate the potential value of including oral and mental health domains in frailty assessment, although external validation in broader and more heterogeneous populations is needed before clinical or screening implementation.

## Supplementary Information

Supplementary Material 1.

## Data Availability

Data may be requested upon reasonable request to the corresponding author.

## Abbreviations

BMI: Body Mass Index
CAAE: Certificado de Apresentação de Apreciação Ética (Brazilian research ethics registry identifier)
CASI: Centro de Apoio e Saúde do Idoso
CES: D—Center for Epidemiologic Studies Depression Scale
CI: Confidence Interval
DA: Discriminant Analysis
DF: Discriminant Function
DF1: Discriminant Function 1
DF2: Discriminant Function 2
EMG: Electromyography
FAT% / FAT (%): Body Fat Percentage
FORP/USP: Faculty of Dentistry of Ribeirão Preto—University of São Paulo
G0 / G1 / G2: Frailty Index groups (G0 Non—frail; G1 Pre—frail; G2 Frail)
G*Power / GPower: G*Power (statistical power analysis software)
GL (DF): Degrees of freedom (as written in the text)
ICC: Intraclass Correlation Coefficient
IPAQ: International Physical Activity Questionnaire
LABIM: Biomechanical and Movement Assessment Laboratory
LANNED: Laboratory of Neuroscience, Neuromodulation and Study of Pain
MCAR: Missing Completely at Random
MANOVA: Multivariate Analysis of Variance
NEUROPAIN: Neuromodulation and Pain Lab
OFI: Oral Frailty Index
SD: Standard Deviation
SPSS: IBM SPSS Statistics
STROBE: Strengthening the Reporting of Observational Studies in Epidemiology
STS: Sit—to—Stand (test)
TUG / TUGT: Timed Up and Go (test)
UNIFAL: MG—Federal University of Alfenas (Minas Gerais)
