# Supplementary material for: Multidimensional frailty assessment integrating physical, mental and oral health: a multicentric study
Source: BMC Oral Health. 2026 Mar 7;26:668. doi: 10.1186/s12903-026-08063-6 (PMC13081566; doi:10.1186/s12903-026-08063-6)

**Supplementary Materials**

Supplementary Table S1. Sex distribution and descriptive statistics for predictor variables (Brazil) (*“Eight‑predictor model”).*

| Fragility group | Sex  (n) | Variable | N | min | max | mean | SD | var |
| --- | --- | --- | --- | --- | --- | --- | --- | --- |
| 0 | F: 22  M: 13 | Right Grip Strength (Kgf) | 35 | 12.0 | 43.9 | 21.4 | 6.7 | 44.7 |
|  |  | TUG (seg) | 35 | 5.0 | 11.5 | 7.9 | 1.8 | 3.2 |
|  |  | FAT (%) | 35 | 16.7 | 49.4 | 32.6 | 8.8 | 77.5 |
|  |  | STS (rep) | 35 | 13.0 | 24.0 | 15.9 | 3.0 | 8.8 |
|  |  | Left Grip Strength (Kgf) | 35 | 10.4 | 35.6 | 20.5 | 6.2 | 38.5 |
|  |  | CES-D | 35 | 0.0 | 15.0 | 6.9 | 4.3 | 18.6 |
|  |  | BMI (kg/m2) | 35 | 19.2 | 38.2 | 27.7 | 4.6 | 20.9 |
|  |  | WHR (w/r) | 35 | 0.6 | 1.1 | 0.9 | 0.1 | 0.02 |
| 1 | F: 95  M: 38 | Right Grip Strength (Kgf) | 133 | 7.7 | 39.8 | 19.2 | 5.9 | 34.3 |
|  |  | TUG (seg) | 133 | 6.0 | 16.8 | 9.8 | 2.2 | 5.0 |
|  |  | FAT (%) | 133 | 5.2 | 60.0 | 36.2 | 11.3 | 128.5 |
|  |  | STS (rep) | 133 | 4.0 | 22.0 | 11.3 | 3.1 | 9.7 |
|  |  | Left Grip Strength (Kgf) | 133 | 8.2 | 39.7 | 18.8 | 6.2 | 38.9 |
|  |  | CES-D | 133 | 0.0 | 37.0 | 9.9 | 7.0 | 48.7 |
|  |  | BMI (kg/m2) | 133 | 15.8 | 44.0 | 28.5 | 5.3 | 28.3 |
|  |  | WHR (w/r) | 133 | 0.4 | 1.1 | 0.9 | 0.1 | 0.0 |
| 2 | F:117  M:10 | Right Grip Strength (Kgf) | 127 | 4.5 | 28.7 | 13.8 | 4.0 | 15.7 |
|  |  | TUG (seg) | 127 | 6.1 | 27.4 | 11.6 | 3.2 | 10.4 |
|  |  | FAT (%) | 127 | 3.0 | 60.0 | 38.4 | 10.9 | 118.9 |
|  |  | STS (rep) | 127 | 5.0 | 15.0 | 9.6 | 2.1 | 4.5 |
|  |  | Left Grip Strength (Kgf) | 127 | 5.8 | 26.4 | 13.5 | 3.9 | 14.9 |
|  |  | CES-D | 127 | 0.0 | 42.0 | 17.7 | 9.8 | 95.7 |
|  |  | BMI (kg/m2) | 127 | 14.6 | 43.1 | 28.4 | 5.4 | 29.5 |
|  |  | WHR (w/r) | 127 | 0.6 | 1.1 | 0.9 | 0.1 | 0.0 |

Supplementary Table S2. Sex distribution and descriptive statistics for predictor variables (Portugal) (*“Eight‑predictor model”).*

| Fragility Group | Sex (n) | Portugal | N | min | max | mean | SD | var |
| --- | --- | --- | --- | --- | --- | --- | --- | --- |
| 0 | F: 49  M: 28 | Right Grip Strength (Kgf) | 77 | 8.4 | 40.2 | 20.0 | 4.9 | 24.3 |
|  |  | TUG (seg) | 77 | 4.5 | 10.2 | 6.9 | 1.2 | 1.5 |
|  |  | FAT (%) | 77 | 16.3 | 43.6 | 32.1 | 6.5 | 42.1 |
|  |  | STS (rep) | 77 | 13.0 | 30.0 | 17.9 | 3.2 | 10.3 |
|  |  | Left Grip Strength (Kgf) | 77 | 8.4 | 33.8 | 19.3 | 4.6 | 21.2 |
|  |  | CES-D | 77 | 0.0 | 18.0 | 7.4 | 4.1 | 16.5 |
|  |  | BMI (kg/m2) | 77 | 18.3 | 39.1 | 26.7 | 4.0 | 15.7 |
|  |  | WHR (w/r) | 77 | 0.7 | 1.1 | 0.9 | 0.1 | 0.0 |
| 1 | F: 62  M: 8 | Right Grip Strength (Kgf) | 70 | 6.8 | 27.5 | 15.5 | 4.3 | 18.7 |
|  |  | TUG (seg) | 70 | 4.4 | 13.9 | 7.6 | 1.9 | 3.8 |
|  |  | FAT (%) | 70 | 15.1 | 48.2 | 35.4 | 6.1 | 36.8 |
|  |  | STS (rep) | 70 | 10.0 | 26.0 | 16.6 | 3.6 | 13.1 |
|  |  | Left Grip Strength (Kgf) | 70 | 7.8 | 25.1 | 15.2 | 4.2 | 18.0 |
|  |  | CES-D | 70 | 1.0 | 28.0 | 11.9 | 6.2 | 38.2 |
|  |  | BMI (kg/m2) | 70 | 18.2 | 40.1 | 27.3 | 4.6 | 21.4 |
|  |  | WHR (w/r) | 70 | 0.8 | 1.2 | 0.9 | 0.1 | 0.0 |
| 2 | F: 56  M: 1 | Right Grip Strength (Kgf) | 57 | 9.0 | 21.6 | 13.4 | 2.8 | 7.7 |
|  |  | TUG (seg) | 57 | 5.3 | 17.8 | 8.5 | 2.2 | 5.0 |
|  |  | FAT (%) | 57 | 7.0 | 52.1 | 36.4 | 5.9 | 35.3 |
|  |  | STS (rep) | 57 | 6.0 | 22.0 | 14.0 | 3.6 | 12.9 |
|  |  | Left Grip Strength (Kgf) | 57 | 5.1 | 18.8 | 13.2 | 2.8 | 8.1 |
|  |  | CES-D | 57 | 2.0 | 42.0 | 23.3 | 9.0 | 81.0 |
|  |  | BMI (kg/m2) | 57 | 20.2 | 45.6 | 28.6 | 4.7 | 21.6 |
|  |  | WHR (w/r) | 57 | 0.7 | 1.1 | 0.9 | 0.1 | 0.0 |

Supplementary Table S3. Univariate normality test (*“Eight‑predictor model”).*

|  | | Shapiro-Wilk test of normality | | | |
| --- | --- | --- | --- | --- | --- |
|  |  | Brazil | | Portugal | |
| Fragility group | Independent variables | df | Sig. | df | Sig. |
| 0 | Right Grip Strength (Kgf) | 35 | 0.001 | 77 | 0.001 |
|  | TUG (seg) | 35 | 0.278 | 77 | 0.191 |
|  | FAT (%) | 35 | 0.387 | 77 | 0.135 |
|  | STS (rep) | 35 | 0.000 | 77 | 0.000 |
|  | Left Grip Strength (Kgf) | 35 | 0.028 | 77 | 0.008 |
|  | CES-D | 35 | 0.159 | 77 | 0.093 |
|  | BMI (kg/m2) | 35 | 0.189 | 77 | 0.031 |
|  | WHR (w/r) | 35 | 0.000 | 77 | 0.500 |
| 1 | Right Grip Strength (Kgf) | 133 | 0.000 | 70 | 0.632 |
|  | TUG (seg) | 133 | 0.000 | 70 | 0.002 |
|  | FAT (%) | 133 | 0.743 | 70 | 0.001 |
|  | STS (rep) | 133 | 0.000 | 70 | 0.180 |
|  | Left Grip Strength (Kgf) | 133 | 0.000 | 70 | 0.098 |
|  | CES-D | 133 | 0.000 | 70 | 0.114 |
|  | BMI (kg/m2) | 133 | 0.910 | 70 | 0.011 |
|  | WHR (w/r) | 133 | 0.000 | 70 | 0.000 |
| 2 | Right Grip Strength (Kgf) | 127 | 0.000 | 57 | 0.103 |
|  | TUG (seg) | 127 | 0.000 | 57 | 0.000 |
|  | FAT (%) | 127 | 0.237 | 57 | 0.000 |
|  | STS (rep) | 127 | 0.001 | 57 | 0.030 |
|  | Left Grip Strength (Kgf) | 127 | 0.027 | 57 | 0.404 |
|  | CES-D | 127 | 0.018 | 57 | 0.281 |
|  | BMI (kg/m2) | 127 | 0.233 | 57 | 0.013 |
|  | WHR (w/r) | 127 | 0.000 | 57 | 0.040 |

Supplementary Table S4. Variance Inflation Factors (VIF) among predictors. using Multiple Regression Models (MRM). for cohort Brazil (*“Eight‑predictor model”).*

|  | Predictors in MRM for multicollinearity assessment | | | | | | | |
| --- | --- | --- | --- | --- | --- | --- | --- | --- |
| Dependent Variable in MRM | Right Grip Strength (Kgf) | TUG (seg) | FAT (%) | STS (rep) | Left Grip Strength (Kgf) | CES-D | BMI (kg/m2) | WHR (w/r) |
| Right Grip Strength (Kgf) |  | 1.771 | 2.237 | 1.668 | 1.296 | 1.093 | 2.128 | 1.039 |
| TUG (seg) | 4.085 |  | 2.238 | 1.045 | 4.064 | 1.081 | 2.113 | 1.038 |
| FAT (%) | 4.061 | 1.761 |  | 1.671 | 3.970 | 1.093 | 1.070 | 1.031 |
| STS (rep) | 4.081 | 1.108 | 2.251 |  | 4.077 | 1.101 | 2.168 | 1.036 |
| Left Grip Strength (Kgf) | 1.298 | 1.765 | 2.190 | 1.669 |  | 1.101 | 2.157 | 1.041 |
| CES-D | 4.057 | 1.739 | 2.235 | 1.670 | 4.079 |  | 2.155 | 1.038 |
| BMI (kg/m2) | 4.009 | 1.725 | 1.110 | 1.669 | 4.054 | 1.094 |  | 1.040 |
| WHR (w/r) | 4.080 | 1.767 | 2.230 | 1.664 | 4.079 | 1.099 | 2.168 |  |

Supplementary Table S5. Variance Inflation Factors (VIF) among predictors. using Multiple Regression Models (MRM). for cohort Portugal (*“Eight‑predictor model”).*

|  | Predictors in MRM for multicollinearity assessment | | | | | | | |
| --- | --- | --- | --- | --- | --- | --- | --- | --- |
| Dependent variable in MRM | Right Grip Strength (Kgf) | TUG (seg) | FAT (%) | STS (rep) | Left Grip Strength (Kgf) | CES-D | BMI (kg/m2) | WHR (w/r) |
| Right Grip Strength (Kgf) |  | 1.685 | 2.047 | 1.562 | 1.541 | 1.142 | 2.713 | 2.096 |
| TUG (seg) | 3.464 |  | 2.011 | 1.123 | 3.750 | 1.140 | 2.601 | 2.099 |
| FAT (%) | 3.471 | 1.658 |  | 1.554 | 3.695 | 1.143 | 2.040 | 2.082 |
| STS (rep) | 3.468 | 1.213 | 2.035 |  | 3.893 | 1.127 | 2.709 | 2.084 |
| Left Grip Strength (Kgf) | 1.375 | 1.628 | 1.945 | 1.565 |  | 1.131 | 2.687 | 2.063 |
| CES-D | 3.473 | 1.687 | 2.050 | 1.545 | 3.854 |  | 2.721 | 2.087 |
| BMI (kg/m2) | 3.457 | 1.612 | 1.533 | 1.555 | 3.837 | 1.140 |  | 1.483 |
| WHR (w/r) | 3.466 | 1.689 | 2.031 | 1.553 | 3.823 | 1.134 | 1.924 |  |

Supplementary Figure 1. Scatter plot of variances versus means of the independent variables. cohort Portugal. (*“Eight‑predictor model”).*


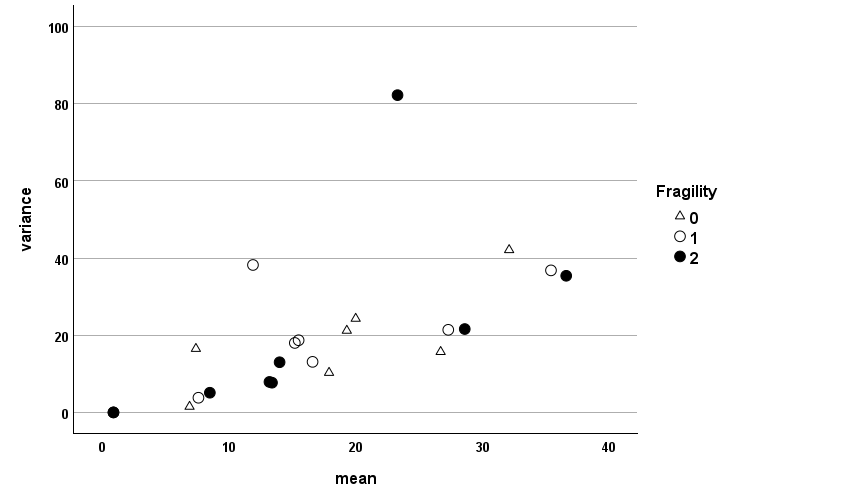


Supplementary Table S6. Sensitivities and Cohen’s k comparison between “Eight-predictor model” and “Ten-predictor model” in both cohorts

| **Cohort** | **Group 0 Sensitivity** | **Group 1 Sensitivity** | **Group 2 Sensitivity** | **Cohen’s κ** |
| --- | --- | --- | --- | --- |
| **Brazil – 8 predictors** | 74.3% (CI: 65.0–82.0) | 66.9% (CI: 58.5–74.3) | 75.6% (CI: 66.0–83.0) | 0.54 (CI: 0.45–0.62) |
| **Brazil – 10 predictors** | 71.4%  (CI: 54.9–83.7) | 64.7%  (CI: 56.2–72.3) | 72.4%  (CI: 64.1–79.5) | 0.53  (CI: 0.46–0.61) |
| **Portugal – 8 predictors** | 68.8%  (CI: 57.8–78.1) | 67.1%  (CI: 58.5–74.3) | 82.5%  (CI: 70.6–90.2) | 0.58  (CI: 0.49–0.66) |
| **Portugal – 10 predictors** | 68.8%  (CI: 57.8–78.1) | 65.7%  (CI: 54.0–75.8) | 82.5%  (CI: 70.6–90.2) | 0.57  (CI: 0.50–0.64) |

| **Multivariate Normality Tests** | | | | | |
| --- | --- | --- | --- | --- | --- |
|  |  | **Brazil** | | **Portugal** | |
| Fragilidade | Test | Statistic | P Value | Statistic | P Value |
| 0 | Henze-Zirkler | 0.969 | 0.312 | 1.08 | <0.001 |
|  | Mardia Skewness | 233.92 | 0.248 | 302.97 | <0.001 |
|  | Mardia Kurtosis | -1.038 | 0.299 | 1.743 | 0.081 |
| 1 | Henze-Zirkler | 1.24 | <0.001 | 1.119 | <0.001 |
|  | Mardia Skewness | 600.39 | <0.001 | 400 | <0.001 |
|  | Mardia Kurtosis | 6.955 | <0.001 | 3.625 | <0.001 |
| 2 | Henze-Zirkler | 1.075 | <0.001 | 1.033 | <0.001 |
|  | Mardia Skewness | 486.41 | <0.001 | 574.64 | <0.001 |
|  | Mardia Kurtosis | 6.411 | <0.001 | 7.07 | <0.001 |

Supplementary Table S7. Multivariate normality tests *(“Ten-predictor model”)*

Supplementary Table S8. Box’s test of Covariance Matrices Homogeneity *(“Ten-predictor model”)*

|  | Covariance Matrices Homogeneity Tests Results | | |
| --- | --- | --- | --- |
|  |  | Brazil | Portugal |
|  | Box's M | 277.93 | 269.7 |
| F | Approx. | 2.336 | 2.277 |
|  | df1 | 110 | 110 |
|  | df2 | 31285 | 97304 |
|  | Sig. | <0.001 | <0.001 |

Supplementary Table S9. Variance Inflation Factors (VIF) among predictors. using Multiple Regression Models (MRM). for cohort Brazil (*“Ten‑predictor model”).*

| Predictors in MRM for multicollinearity assessment | | | | | | | | | | |
| --- | --- | --- | --- | --- | --- | --- | --- | --- | --- | --- |
|  | BMI (kg/m2) | FAT (%) | WHR (w/r) | Left Grip Strength (Kgf) | Rigth Grip Strength (Kgf) | STS (rep) | TUG (seg) | CES-D | Bite Strength (Kgf) | OFI-8/PT |
| BMI (kg/m2) |  | 1.141 | 1.043 | 4.070 | 4.116 | 1.681 | 1.774 | 1.148 | 1.254 | 1.249 |
| FAT (%) | 1.079 |  | 1.033 | 3.977 | 4.166 | 1.684 | 1.810 | 1.151 | 1.243 | 1.245 |
| WHR (w/r) | 2.199 | 2.303 |  | 4.098 | 4.172 | 1.678 | 1.829 | 1.155 | 1.271 | 1.247 |
| Left Grip Strength (Kgf) | 2.185 | 2.260 | 1.044 |  | 1.343 | 1.683 | 1.822 | 1.156 | 1.270 | 1.246 |
| Rigth Grip Strength (Kgf) | 2.168 | 2.323 | 1.043 | 1.317 |  | 1.680 | 1.831 | 1.151 | 1.252 | 1.247 |
| STS (rep) | 2.196 | 2.328 | 1.040 | 4.094 | 4.167 |  | 1.145 | 1.156 | 1.261 | 1.248 |
| TUG (seg) | 2.132 | 2.302 | 1.042 | 4.077 | 4.176 | 1.053 |  | 1.142 | 1.246 | 1.243 |
| CES-D | 2.185 | 2.319 | 1.043 | 4.097 | 4.156 | 1.684 | 1.809 |  | 1.269 | 1.191 |
| Bite Strength (Kgf) | 2.170 | 2.277 | 1.044 | 4.094 | 4.113 | 1.671 | 1.795 | 1.155 |  | 1.144 |
| OFI-8/PT | 2.199 | 2.320 | 1.042 | 4.088 | 4.170 | 1.683 | 1.823 | 1.102 | 1.164 |  |

Supplementary Table S10. Variance Inflation Factors (VIF) among predictors. using Multiple Regression Models (MRM). for cohort Portugal (*“Ten‑predictor model”).*

| Predictors in MRM for multicollinearity assessment | | | | | | | | | | |
| --- | --- | --- | --- | --- | --- | --- | --- | --- | --- | --- |
|  | BMI (kg/m2) | FAT (%) | WHR (w/r) | Left Grip Strength (Kgf) | Rigth Grip Strength (Kgf) | STS (rep) | TUG (seg) | CES-D | Bite Strength (Kgf) | OFI-8/PT |
| BMI (kg/m2) |  | 1.542 | 1.494 | 3.879 | 3.525 | 1.608 | 1.632 | 1.209 | 1.199 | 1.291 |
| FAT (%) | 2.052 |  | 2.094 | 3.739 | 3.541 | 1.605 | 1.683 | 1.213 | 1.201 | 1.288 |
| WHR (w/r) | 1.935 | 2.037 |  | 3.856 | 3.540 | 1.602 | 1.713 | 1.206 | 1.198 | 1.287 |
| Left Grip Strength (Kgf) | 2.698 | 1.954 | 2.071 |  | 1.478 | 1.619 | 1.658 | 1.203 | 1.190 | 1.290 |
| Rigth Grip Strength (Kgf) | 2.719 | 2.052 | 2.108 | 1.639 |  | 1.617 | 1.706 | 1.213 | 1.199 | 1.271 |
| STS (rep) | 2.716 | 2.038 | 2.090 | 3.933 | 3.542 |  | 1.239 | 1.200 | 1.171 | 1.274 |
| TUG (seg) | 2.603 | 2.018 | 2.110 | 3.802 | 3.529 | 1.170 |  | 1.213 | 1.192 | 1.287 |
| CES-D | 2.726 | 2.055 | 2.100 | 3.899 | 3.548 | 1.602 | 1.714 |  | 1.192 | 1.240 |
| Bite Strength (Kgf) | 1.236 | 2.731 | 2.055 | 2.107 | 3.896 | 3.542 | 1.578 | 1.701 |  | 1.203 |
| OFI-8/PT | 2.734 | 2.050 | 2.106 | 3.929 | 3.492 | 1.597 | 1.709 | 1.164 | 1.149 |  |

Supplementary Figure 2. Scatter plot of variances versus means of the independent variables. cohort Brazil *(“Ten-predictor model”)*
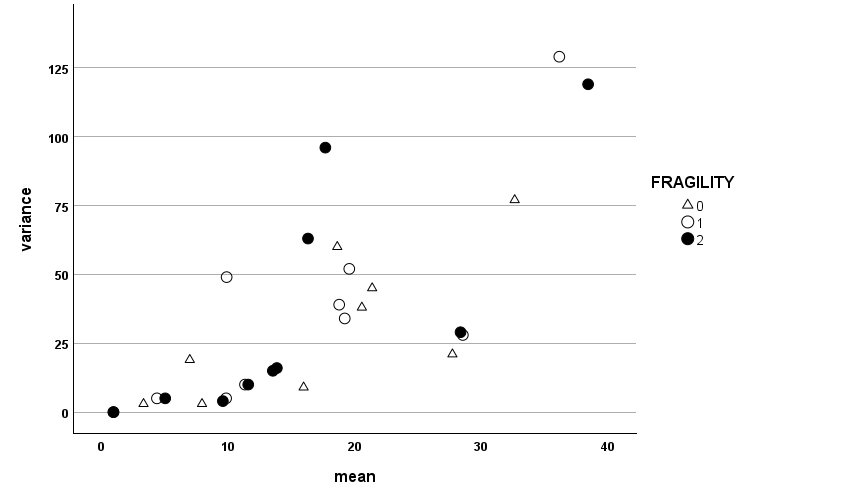


Supplementary Figure 3. Scatter plot of variances versus means of the independent variables. cohort Portugal *(“Ten-predictor model”)*


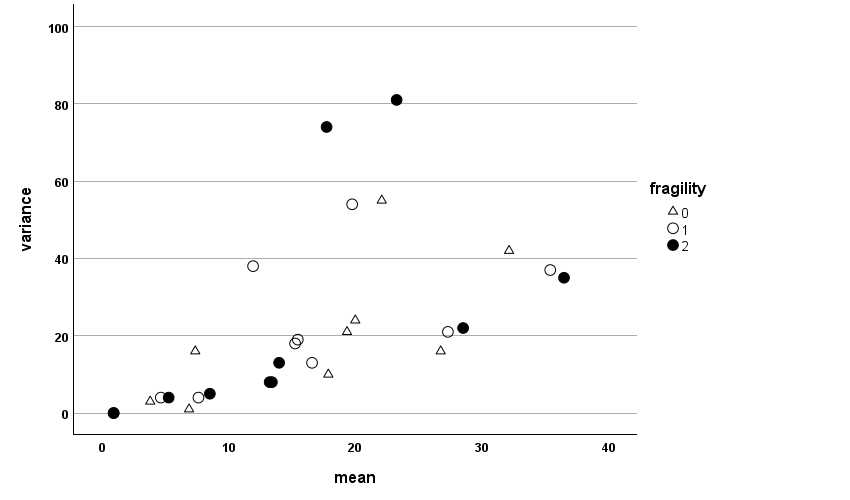

Supplement: Supplementary file 1 — Supplementary Material 1. [file 12903_2026_8063_MOESM1_ESM.docx]
